# Modeling Textural Properties of Cooked Germinated Brown Rice Using the near-Infrared Spectra of Whole Grain

**DOI:** 10.3390/foods12244516

**Published:** 2023-12-18

**Authors:** Kannapot Kaewsorn, Thitima Phanomsophon, Pisut Maichoon, Dharma Raj Pokhrel, Pimpen Pornchaloempong, Warawut Krusong, Panmanas Sirisomboon, Munehiro Tanaka, Takayuki Kojima

**Affiliations:** 1 Department of Agricultural Engineering, School of Engineering and Innovation, Rajamangala University of Technology Tawan-Ok, Chon Buri 20110, Thailand; kannapot_ka@rmutto.ac.th; 2 Department of Agricultural Engineering, School of Engineering, King Mongkut’s Institute of Technology Ladkrabang, Bangkok 10520, Thailand; thitimap.june@gmail.com (T.P.); palmpisut1994@gmail.com (P.M.); 64601186@kmitl.ac.th (D.R.P.); 3 Department of Food Engineering, School of Engineering, King Mongkut’s Institute of Technology Ladkrabang, Bangkok 10520, Thailand; pimpen.po@kmitl.ac.th; 4 Division of Fermentation Technology, School of Food Industry, King Mongkut’s Institute of Technology Ladkrabang, Bangkok 10520, Thailand; warawut.kr@kmitl.ac.th; 5 Laboratory of Agricultural Production Engineering, Faculty of Agriculture, Saga University, 1 Honjo-machi, Saga 840-8502, Japan; kojimat1733@gmail.com

**Keywords:** germinated brown rice, hardness, toughness, texture, near-infrared spectroscopy, machine learning, partial least squares regression, artificial neural network

## Abstract

If a non-destructive and rapid technique to determine the textural properties of cooked germinated brown rice (GBR) was developed, it would hold immense potential for the enhancement of the quality control process in large-scale commercial rice production. We combined the Fourier transform near-infrared (NIR) spectral data of uncooked whole grain GBR with partial least squares (PLS) regression and an artificial neural network (ANN) for an evaluation of the textural properties of cooked germinated brown rice (GBR); in addition, data separation and spectral pretreatment methods were investigated. The ANN was outperformed in the evaluation of hardness by a back extrusion test of cooked GBR using the smoothing combined with the standard normal variate pretreated NIR spectra of 188 whole grain samples in the range of 4000–12,500 cm^−1^. The calibration sample set was separated from the prediction set by the Kennard–Stone method. The best ANN model for hardness, toughness, and adhesiveness provided R^2^, r^2^, RMSEC, RMSEP, Bias, and RPD values of 1.00, 0.94, 0.10 N, 0.77 N, 0.02 N, and 4.3; 1.00, 0.92, 1.40 Nmm, 9.98 Nmm, 1.6 Nmm, and 3.5; and 0.97, 0.91, 1.35 Nmm, 2.63 Nmm, −0.08 Nmm, and 3.4, respectively. The PLS regression of the 64-sample KDML GBR group and the 64-sample GBR group of various varieties provided the optimized models for the hardness of the former and the toughness of the latter. The hardness model was developed by using 5446.3–7506 and 4242.9–4605.4 cm^−1^, which included the amylose vibration band at 6834.0 cm^−1^, while the toughness model was from 6094.3 to 9403.8 cm^−1^ and included the 6834.0 and 8316.0 cm^−1^ vibration bands of amylose, which influenced the texture of the cooked rice. The PLS regression models for hardness and toughness had the r^2^ values of 0.85 and 0.82 and the RPDs of 2.9 and 2.4, respectively. The ANN model for the hardness, toughness, and adhesiveness of cooked GBR could be implemented for practical use in GBR production factories for product formulation and quality assurance and for further updating using more samples and several brands to obtain the robust models.

## 1. Introduction

Rice (*Oryza Sativa* L.), as the world’s primary staple food, has a critical role to play in providing 20% of the calorie intake for nearly half of the global population [1,2]. This essential crop accounts for 19% of dietary energy worldwide [3,4]. Among the leading rice-exporting nations, Thailand, alongside India and Vietnam, stands as a consistent top performer in the global rice export sector [5]. Projections for 2023 indicated a further increase, with anticipated Thai rice exports reaching 8.3 million metric tons [6]. This not only bolsters Thailand’s economic prosperity but also solidifies its historical moniker as the “Rice Bowl of the World”.

Thailand cultivates a wide variety of rice, with Jasmine rice (Thai Hom Mali) being the most famous [7,8]. Other varieties include glutinous rice, black rice (riceberry), red cargo rice, and various fragrant and non-fragrant rice types. Brown rice is a whole grain rice variety that is minimally processed and retains its outer bran layer and germ.

As Thai consumers become increasingly health-conscious, there is a growing preference for foods that offer health benefits [9]. As with many other countries, Thailand faces a rising burden of non-communicable diseases (NCDs), such as obesity, diabetes, and cardiovascular disorders [10]. Germinated brown rice (GBR) has the potential to mitigate these health issues, owing to its low glycemic index and antioxidant properties, which provide health benefits such as blood pressure reduction, sleepiness improvement, cardiovascular disease reduction, and diabetes regulation, and it may limit weight gain [11,12]. Thus, GBR is positioned to be a valuable dietary component in the battle against NCDs. As the global demand for healthy and specialty foods continues to grow, Thai GBR has become an export commodity. Its unique nutritional attributes make it an appealing product for international markets; thus, it contributes to Thailand’s agricultural exports and economy.

If a non-destructive and rapid technique to determine the textural properties of cooked GBR was developed, it would hold immense potential for the enhancement of the quality control process in large-scale commercial rice production.

The texture of cooked rice plays a pivotal role in defining the palatability and overall dining experience [13]; texture refers to the physical feel and structure of the rice grains, including attributes like firmness, chewiness, stickiness, and grain separation [14], which determine the market value and become the driving factors in consumer preferences for rice. However, the consumption of brown rice is limited, and some of the barriers include the perceptions of the rough texture and unpalatable taste and the increased length of time for cooking [15].

A number of methods have been employed to improve the textural properties of brown rice, e.g., soaking, gamma radiation, ultrasonic treatment, enzyme treatment, high-pressure cooking, freeze–thaw cycle treatment, and germination [15]; a germination level of 70% is considered to be the minimum that is required to produce GBR [16]. Germinated brown rice, often referred to as GBR or GABA rice, is also simply called brown rice that has undergone a natural germination process [17]. The germination process activates Though they did not consider the NIR whole grain spectra of Indica rice but rather the spectra of its cooked rice, PLSR models were developed in 2007 which predicted sensory hardness and stickiness slightly better than the glossiness, with *r*^2^_v_ values ranging from 0.88 to 0.91 [18]. enzymes such as α-amylose, proteas, phytase, and lipase within the rice grains, leading to various changes in the rice’s nutritional content and flavor profile, and resulting in softer and sweeter cooked brown rice [19,20]. Because the bran layer contributes to the hard chewy texture usually favored by consumers, increased softness is an important attribute of the eating quality of cooked GBR [21].

Recent research has advanced non-destructive techniques using NIR radiation combined with machine learning (ML) to evaluate rice quality, including texture. Machine learning (ML) can be used to model such predictive relationships based on NIR spectra; it is commonly used with principal component regression (PCR), support vector machine regression (SVR), partial least squares regression (PLSR) and artificial neural network (ANN) algorithms. PLSR and PCR were combined with the NIR spectra for the evaluation of the hardness and toughness of cooked parboiled rice, respectively [22]; however, the models were only applicable for screening and approximate calibration. The artificial neural networks (ANNs) combined with the NIR spectra of raw rice provided a model with a high R = 0.94; this combination could estimate the color, texture, and pH of cooked rice, offering a rapid and cost-effective method [23]. Together, these findings highlight the potential of digital technologies, artificial intelligence, and spectroscopy to enhance rice quality assessment efficiently and precisely. For other food, PCR, SVR, PLSR, and BP-ANN combined with NIR spectra were used in the prediction of the quality indicators of frozen samples, such as drip loss and texture parameters, including hardness, chewiness, gumminess, and gel strength, respectively; by comparison, the BP-ANN modeling approach performed better than the others [24].

The objective of this study is to develop a predictive model for assessing the textural properties, including the hardness, toughness, stickiness, and adhesiveness of cooked GBR, solely from the near-infrared spectra of uncooked GBR grains; the aim is to offer a nondestructive and efficient method for quality control in GBR production.

In this study, two algorithms, PLSR and an ANN, were used to develop the models. PLSR is a traditional statistical method; it is a multivariate technique that constructs latent variables and their factors or components to capture the maximum covariance between the predictors and response variables. PLSR is particularly effective as a linear algorithm with high-dimensional datasets with multicollinearity. An ANN is a hybrid algorithm which can deal with linear and non-linear datasets. It can be considered for use as an ML algorithm for a small-sized dataset or a highly advanced deep learning algorithm for a big dataset.

To date, there has been no report on the evaluation of the textural properties of cooked germinated brown rice using the near-infrared spectra of uncooked whole grains.

## 2. Materials and Methods

### 2.1. Rice Samples

Rough rice of *Oryza sativa* L., cultivar Khao Dawk Mali 105 (KDML 105) was collected from a field of P.J. Brand germinated rough rice factory in Chonburi Province, Thailand. The GBR was created using the technique described by Kaewsorn and Sirisomboon [25] and Kaewsorn et al. [26]: the rough rice water soaking times at room temperature were 24 and 48 h, and seven different incubation intervals (0, 6, 12, 18, 24, 30, and 36 h) to create the germinated rough rice (GRR) were used. The GRR was dried using the fluidized-bed process and the air-dried process. Prior to the experiment, the GRR sample was dehusked and is referred to as GBR in this paper. Each treatment condition employed ten kg of GBR. There was 1 control condition (regular brown rice, 0 h soaking time, and 0 h incubation time) and 14 treatments with 2 replicates, resulting in 30 samples used. The 32 commercial types and brands of GBR of the various varieties and some of the same varieties indicated were purchased from local department stores in Bangkok, Thailand, and stored at room temperature in the laboratory. The commercial GBR for the 32 brands with different varieties was specified in Kaewsorn et al. [26]. There were 16 brands for the KDML and 105 varieties (2 replicates and 32 samples) and 16 brands for the other variations (2 replicates and 32 samples). As a result, 64 samples from local marketplaces were obtained.

### 2.2. GBR Uncooked Sample Scanning for NIR Spectra

The FT-NIR spectrometer was used in this experiment. It has some advantages over the grating NIR spectrometer, including: (1) higher signal-to-noise ratios; (2) extremely high resolutions; and (3) fast and accurate frequency determinations [27,28]. FT-NIR spectroscopy was successfully applied to analyze the properties of rice, such as the lipid content of milled rice (long, medium, and short grains) [28,29], the gamma oryzanol of GBR [25], and the optimal cooking time of rice [29,30].

Each sample was emptied from a vacuum bag into the quartz bottom-sampling cup (87 mm diameter and 87.5 mm height) placed in the rotational diffuse reflectance holder of Bruker Ltd. (Ettlingen, Germany). The NIR spectra were measured in diffuse reflection mode with an FT-NIR spectrometer (Bruker Ltd., Ettlingen, Germany) at a wavenumber of 4000–12,500 cm^−1^ (800–2500 nm). At a resolution of 16 cm^−1^, each rice sample was scanned 64 times. In the absorption mode (log 1/R), the scan findings were averaged and recorded. Prior to future usage, the quartz bottom-sampling cup was vacuum-cleaned. The background compensation was conducted before the scanning of each sample by the internal scanning of gold plate as a reference material. The scanning was conducted in a 25 ± 2 °C air-conditioned room.

### 2.3. The Approximate Repeatability of NIR Scanning

The scanning was conducted twice per sub-sample at the same location, and there were 2 sub-samples per sample. The standard deviation of the absorption value at each wavenumber of every sub-sample was calculated, and the values of every sample were averaged. Then, the values of every wavenumber were averaged to obtain the approximate repeatability of the NIR scanning. The genuine repeatability can be obtained by scanning the sample at the same location at least 10 times continuously [31].

### 2.4. Method of Cooking Rice

The rice-cooking technique utilized by Sirisomboon et al. [32] involved the use of personal rice cookers (RC−10 MM, Toshiba, Bangkok, Thailand) to prepare 200 g of GBR samples using water-to-rice ratios of 1.6:1. To produce cooked rice with the customary texture that customers want, the required water-to-rice ratio was employed. The cooked GBR was placed in a plastic cup with a weight of approximately 5 g. In total, 5 cups per sample were prepared.

This rice-cooking method is described in detail by Reyes and Jindal [33], Srisawas and Jindal [34], and Parnsakhorn and Noomhorm [35]; it is a reliable method for cooking KMDL rice.

### 2.5. Back Extrusion Test for Texture of Cooked GBR

The back extrusion (BE) test rig developed by researchers in the Asian Institute of Technology has been used for measuring the hardness of cooked rice [33,36,37], and the BE instrument could best interpret the sensory hardness–softness texture of cooked rice [36]. The BE testing of the textural properties of cooked GBR rice showed a high measurement precision in the hardness, toughness, and stickiness tests, respectively [23]. Therefore, we used the BE for our experiment.

The cooked GBR samples were next subjected to the back extrusion test, following the method of Kaewsorn et al. [26], which involved inserting 3 g of cooked rice into a back extrusion test rig (BE) that was pressured from the top entrance of the rice container by a stainless ball for 99 mm of the total height of 100 mm, at a ball probe speed of 1 mm/s. Each sample’s mean was calculated using 5 duplicate tests. The hardness, toughness, stickiness, and adhesiveness of the cooked GBR was determined. The back extrusion test was performed on 94 samples, with the average of each sample obtained using 5 replications.

### 2.6. The Repeatability and Reproducibility of the Measurement of Textural Properties

The repeatability and reproducibility of the measurement of the textural properties were determined by measuring four duplicates (four pairs) that were randomly subjected during the experiment at different times. These were reported in Kaewsorn et al. [26].

### 2.7. NIR Spectroscopy Modeling by Machine Learning

#### 2.7.1. Calibration Set and Prediction Set Separation

To check the model performance, several methods of sample division for the calibration and prediction sets were employed as this significantly impacts the model performance [38]. The calibration set should contain enough representative information to model unknown samples in the future [39]; it should be the largest among them and should have validation data coverage. If the calibration set’s value range does not adequately cover the validation set, prediction errors may occur because the model has not seen data with higher or lower values. The validation set is essential for effectively evaluating the model [40]. In this study, we focused on four methods for sampling, with an 80% calibration set and a 20% validation set: interval sampling (IS), Kennard–Stone (KS), hold-out cross-validation (hold-out CV), and sorting.

The IS method was obtained by selecting validation samples using the following steps: (1) the samples were sorted into ascending or descending order according to the reference value; (2) the samples were divided into subsets, with each subset containing five samples; (3) the middle sample in each subset was selected to be the validation data [41].

The KS method involves selecting samples that are uniformly distributed based on Euclidean distance for distance computations [42]. This method is implemented using the following steps: (1) find the sample that is closest to the mean of the samples to be used as validation data and remove it from the dataset; (2) find the sample that is the most dissimilar to the sample selected in step (1) to be used as validation data and remove it from the dataset; (3) find the sample that is the most dissimilar to the samples that have already been allocated to the validation set based on the minimum distance from any sample allocated to be validation data and remove this sample from the dataset; (4) repeat step (3) until the desired amount is reached [43].

The CV method using a hold-out strategy involved random sampling without considering the data distribution. The proportions of the data split could vary, ranging from 90%:10% to 80%:20%, creating two mutually exclusive datasets: the training (calibration) dataset and the test (validation) dataset [44].

The last method is sorting, which is similar to the IS method. It involves dividing the samples into subsets (with each subset containing ten samples) and then selecting the seventh to eighth samples in each subset to be the validation data.

All four sampling methods provided different information for the calibration and validation sets, including sample distribution in each dataset (Supplementary Data S1). To determine which method is the most suitable for our data and yields the best predictions, a comparison is needed.

#### 2.7.2. Spectral Pretreatment

The spectral interferences are shown by a combination of several additive factors, multiplicative factors, polynomial baseline shifts, and spectral noises; hence, the empirical methods are widely used for spectral preprocessing [45]. Naturally, the raw spectrum may contain noise due to factors such as sample size [46] or moisture [47] that affects the light scattering [48]. This issue can be effectively addressed through spectral pretreatment. Pretreatment techniques play a crucial role in various analytical and data applications as they serve to enhance the quality of the data before further analysis is conducted [49].

For modeling by OPUS, v. 7.0, (Bruker, Ettlingen, Germany), the following pretreatment algorithms were used in both the spectrum pretreatment and the model development. The NIR absorption spectra were combined with the reference data. After sorting the texture data, the entire spectra data were divided into calibration and prediction sets with a 7:3 ratio. The NIR spectra used for the model development were not preprocessing, constant offset elimination, straight line subtraction, vector normalization (SNV), min−max normalization, multiplicative scatter correction (MSC), first derivatives, second derivatives, first derivatives + straight line subtraction, first derivatives + SNV, or first derivatives + MSC.

In the case of MATLAB, the spectral pretreatment was conducted with no pretreatment when the abbreviation was Raw—raw spectrum, and when there was pretreatment applied, the methods included: BL—baseline offset spectrum; MC—mean centering spectrum; MN—mean normalization spectrum; MMN—max–min normalization spectrum; SMT—smoothing spectrum; SMT + SNV—smoothing + standard normal variate spectrum; SMT + MSC—smoothing + multiplicative scatter correction spectrum; SMT + 1D—smoothing + 1st derivative spectrum; and SMT + 2D—smoothing + 2nd derivative spectrum spectrometer.

The mean centering transformation, which is the mean of the absorption values of every sample spectrum in each wave band in the spectral data matrix, is subtracted from each value in that wave band; hence, the mean centering centers the values corresponding to each band about zero (modified [50]). The mean centering amplifies the differences between the sample spectra [51]. The mean normalization and max−min normalization normalize the spectra so that they have a common feature by dividing each absorption value of each band in the raw spectrum by the average absorption value and the range (subtracting the maximum value from the minimum value) absorption value, respectively, of the spectrum. The normalization pretreatment corrects the spectral change caused by small light path differences [51].

The baseline offset correction only removes the baseline shift, where every band absorbance of each spectrum is corrected by subtracting either its absorbance at the first band (or another arbitrarily chosen band) or the median value in a selected range of spectra [52].

The standard normal variate (SNV) method was employed as a pretreatment step before modeling. SNV operates by centering each spectrum around zero, which is achieved by subtracting the mean and then scaling each signal value by the standard deviation of the entire spectrum. SNV is highly effective in removing systematic variations in spectral data, rendering it well-suited for subsequent analyses [53].

The spectra are shifted linearly so that the minimum y−value is equal to zero for the pretreatment of the constant offset elimination, in order to eliminate the linear baseline shifts; this is also conducted by the subtraction of a straight line pretreatment; in each selected frequency range in the spectrum, the straight line is fitted by the partial least squares method; then, the straight line is subtracted from the respective spectrum to eliminate the linear tilt of the baseline shift [54]. Min−Max−Normalization (for absorbance spectra): The spectra are shifted linearly so that the minimum Y−value equals zero; then, the spectra are expanded so that the maximum Y−value equals two absorbance units; this spectral pretreatment can eliminate the influence of the optical path length in the changing height of the signal but not its structure in the transmission mode, while in the diffuse reflectance mode the effect of different density or different particle sizes can often be minimized [55]. The first derivative spectral pretreatment is conducted by taking the derivative of each gap consecutively along the raw spectrum, and if another derivative is taken on the first derivative spectrum, then it is the second derivative pretreatment spectrum. By these pretreatment methods, the baseline of every sample spectrum is the same baseline (common baseline), and the baseline shift is eliminated. The first derivative makes the peak of the raw spectrum become a zero-intensity point in the pretreated spectrum, and the slope change point in the raw spectra will be the peak of the first derivative spectrum. The second derivative pretreatment shows the peaks and the overlapping peaks, but upside down, whereas the NIR radiation absorption by the corresponding bond vibration is better shown. The slope change points along the raw spectra will be the peaks of the shoulders of the second derivative spectra. These pretreatments can make the absorption at the amplified peaks correlate well with the dependent variables; then, the model performance is improved. The smoothing pretreatment is needed to suppress noise before derivation if the raw spectrum has a noise signal which is in the spike form. However, in the presence of complex interferences or when inappropriate smoothing parameters are used, the result of the derivation may be rendered ineffective [45].

Multiplicative scattering correction (MSC) is used to compensate for additive and multiplicative effects in the spectral data [52]. The effects are caused by the physical differences of the samples, such as different particle sizes, fruit sizes, and/or the density of the samples and the uncertainty of the spectrometer due to a change in humidity and temperature. The MSC spectrum is obtained by the linear relationship calculated by ordinary least squares regression between the absorbance of the average spectrum of the calibration sample spectra and that of the sample raw spectrum; the additive factor and multiplicative factor are calculated for the treatment of the raw spectrum to transform it into the MSC spectrum, and the factors are saved for the treatment of the prediction sample set spectra.

#### 2.7.3. Modeling Algorithms

##### Partial Least Squares Regression

Partial least squares (PLS) regression is a chemometric algorithm used for modeling to predict dependent variables (Y, in this work, is texture) from independent variables (X, in this work, is the NIR spectra); this is helpful in evaluating the data of both X and Y with large, noisy, collinear, and even missing variables [55]. PLS was based on principal component regression (PCR), but PLS created new variables called latent variables (LVs), which are combined with the regression [56]. The LVs are linear combinations of the original independent variables and are constructed in a way that maximizes the covariance between the independent and the response variable [57].

In this study, PLS regression was employed to predict the texture (quantification) of cooked GBR by using the spectra of GBR grains; OPUS software version: 7.8 (Bruker, Germany) and by MATLAB version: 9.13.0 (R2022b) were used [58]. In the MATLAB calculation, LVs from 1 to 20 were used for modeling to compare their performances.

##### Artificial Neural Network

Artificial neural networks (ANNs) work in a similar way to the human nervous system when they train the independent variables to describe the dependent variables [59]. The ANN is a nonlinear model that can be used to handle complicated relationships for classification [60]. It is based on a supervised procedure and consists of input (X), hidden, and output (Y) layers with connected neurons (nodes) to simulate the network and compute weights/bias trade-offs [61,62].

In this work, the hidden layers of (5, 10); (10, 10); (15, 10); (20, 10); (25, 10); and (30, 10) were used for modeling. Every set of hidden layers was generated 20 times to find the best-performing model. The modeling was performed by MATLAB version: 9.13.0 (R2022b) [58].

#### 2.7.4. Model Performance Determination

The prediction performance of the model was evaluated by the error (*e*) that occurred in each prediction of sample *i*; *i* was equal to 1 to *n*, which was calculated by subtracting the reference measured value (*y*) from the NIR predicted value (*ŷ*) of either the calibration set or the prediction set. The averaged value of *y_i_* (*ӯ*) was used together with *y_i_* and *ӯ* to calculate the coefficient of determination.

The coefficient of determination of the calibration (R^2^) and of the prediction (r^2^) were calculated by Equation (1):

(1) $$R^{2}\;\mathrm{or}\;r^{2}=1-\frac{{\sum_{i=1}^{n}{(y}_{i}-{ŷ}_{i})}^{2}}{{\sum_{i=1}^{n}{(y}_{i}-ȳ)}^{2}}$$

where the model should be improved.

The root mean square errors (RMSEs) of calibration (RMSEC) and of prediction (RMSEP) were calculated by Equation (2):

(2) $$\mathrm{RMSE}=\sqrt{\frac{{\sum_{i=1}^{n}{(y}_{i}-{ŷ}_{i})}^{2}}{n}\;}=\sqrt{\frac{{\sum_{i=1}^{n}e_{i}}^{2}}{n}\;}$$

The bias was calculated by Equation (3):

(3) $$\mathrm{Bias}=\frac{\sum_{i=1}^{n}\left(y_{i}-{ŷ}_{i}\right)}{n}=\frac{\sum_{i=1}^{n}e_{i}}{n}$$

The ratio of prediction to deviation (RPD) was calculated by dividing the standard deviation of the prediction set (SD) by the RMSEP in Equation (4):

(4) $$\mathrm{RPD}=\frac{SD}{RMSEP}$$

## 3. Results and Discussion

### 3.1. Spectral Characteristic of Whole Grain GBR

**Figure 1:**
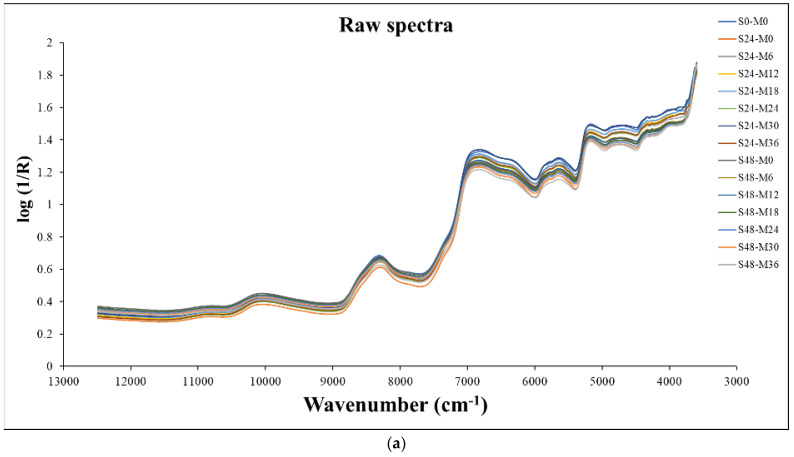

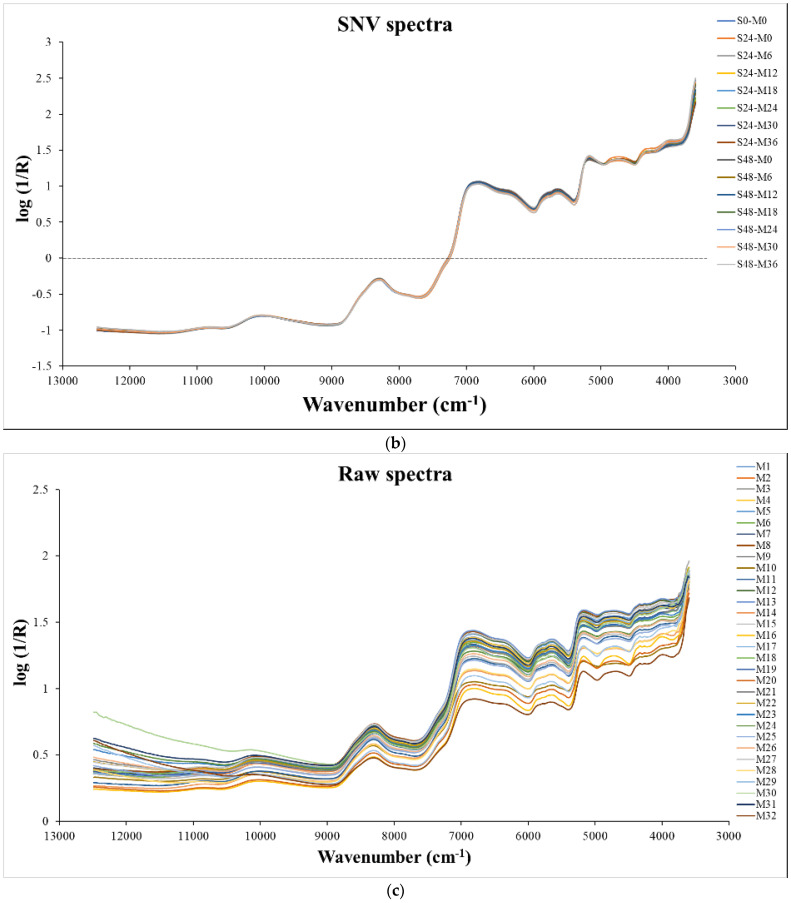

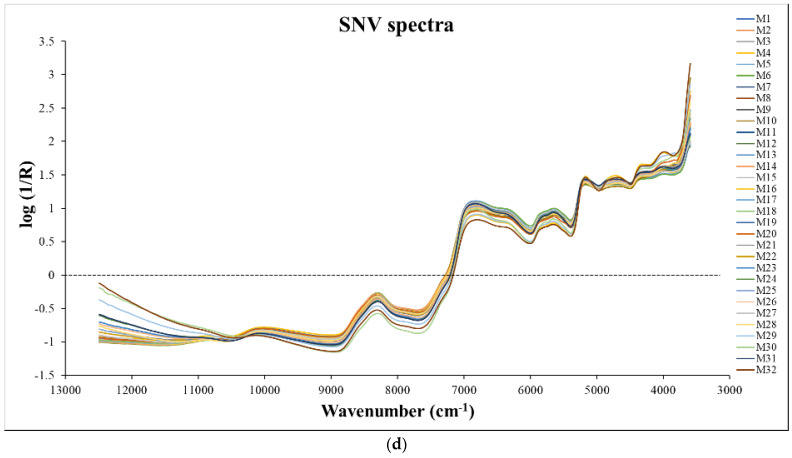
(**a**) The raw spectra; (**b**) standard normal variate (SNV) pretreated spectra of GBR grain samples in different conditions of germinating processes; (**c**) the raw spectra; (**d**) SNV pretreated spectra of commercial GRB bought from markets.

Figure 1a,b show the raw spectra and standard normal variate (SNV) pretreated spectra, respectively, of the GBR grain samples in the different conditions of the germinating processes; the structure of the spectra was same as that of the raw spectra and the SNV pretreated spectra, respectively, of the commercial GRB bought from the markets, as shown in Figure 1c,d. It was obvious that the GBR spectra from the different conditions of the germinating processes showed less baseline shift than the spectra of the commercial GBR, even with the SNV pretreatment; this is obviously due to different production protocols, which create the differences in the constituents and the matrix of the GBR.

The average raw NIR spectra of the germinated brown rice acquired throughout the wave number range of 4000–12,500 cm^−1^ appeared in the peaks at 10,013, 8262, 6781, 6333, 5763, 5608, and 5161 cm^−1^. The peak at 10,013 cm^−1^ (about 990 nm) corresponds to the absorption band of the second overtone associated with the starch’s O–H stretching. The peak at 8262 cm^−1^ (1210 nm) relates to the second overtone associated with the CH_2_ group’s C–H stretching (usually found around 1215 nm); at 6333 cm^−1^ (1579 nm) due to the absorption band associated with the first overtone of the C–H stretching of starch (1580 nm); at 5608 cm^−1^ (1783 nm) due to the first overtone of the C–H stretching of cellulose (typically found at 1780 nm); and at 5161 cm^−1^ (1938 nm) due to a combination of vibrations that were due to the O–H stretching + the O–H deformation of water [63]. Furthermore, the Savizky−Golay second derivative spectra of the GBR samples show the CH_3_, CH_2_, CO_2_H, and cellulose [63] (Figure 1c).

### 3.2. Overall Precision Test

**Figure 2:**
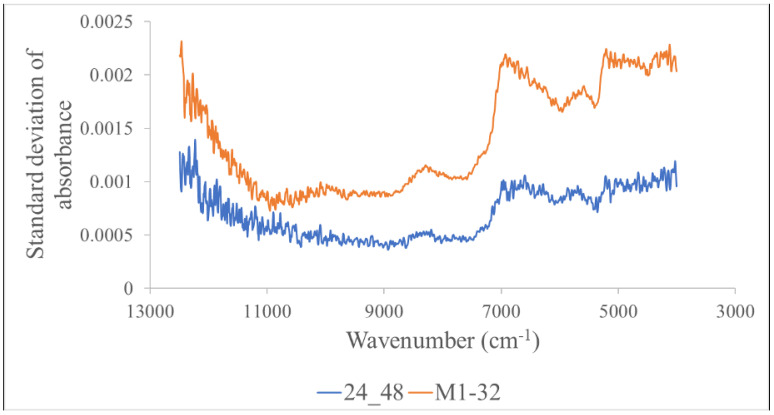
Standard deviation graph of absorbance of condition-adjusted GBR samples (blue) and commercial samples (red) changed with wavenumber.

The spectral precision levels of the condition-adjusted GBR and the commercial GBR of the 32 brands, as indicated by the average standard deviation of the absorption values of every wavenumber, were 0.00154 and 0.00080, respectively. The spectral precision levels of the whole wheat grains scanned by the FT–NIR spectrometer, as reported by [31], were 0.00310, 0.0034, 0.00494, and 0.00782 at 10,373.4, 8665.5, 8333.3, 5896.3, and 4262.6 cm^−1^, respectively. Figure 2 shows the different levels of repeatability with the reduction in wavenumber; the beginning and end show low repeatability, and the middle shows higher repeatability, though the pattern of the two GBR groups was the same.

The repeatability and reproducibility of the texture measurements of the cooked GBR that were reported by Kaewsorn et al. [26] for the same sample sets used in this experiment were 1.31 and 1.42 N, 13.97 and 13.34 Nmm, 0.83 and 0.38 N, and 2.87 and 12.79 Nmm for hardness, toughness, stickiness, and adhesiveness, respectively. These values provided the maximum R^2^ for the NIR predictions of 0.84, 0.83, 0.46, and 0.92 for hardness, toughness, stickiness, and adhesiveness, respectively; the calculation was made using the method used by Sirisomboon and Nawayon [64], Pornchaloempong et al. [65], and Lim and Sirisomboon [66] and the statistics of the calibration set obtained by the KS method. These maximum R^2^ values can be obtained when there is no NIR error but there is a reference laboratory error. This indicated that the back extrusion test for the stickiness must be researched with regard to why high error was obtained or why there was variation among the samples with a low standard deviation of stickiness.

### 3.3. Prediction Performance of PLS Regression Model for Texture of Cooked GBR by Uncooked GBR Grains by OPUS

The minimum (min), maximum (max), mean and standard deviation (SD) of the textural properties of cooked GBR used for modeling by OPUS and MATLAB are shown in Table 1 and Table 2. From Table 3, it is obvious that the prediction using total samples for the textural properties was poor, with r^2^ being only 0.21–0.63, which was the same as for the GBR production condition-adjusted samples and the 32 commercial brand samples, where there were r^2^ values of 0.03–0.92 and 0.44–0.71, respectively, but with underfitting prediction. However, when the commercial brand samples were separated into the KDML GRB group and the various variety groups, the model performance was better, but only for the hardness of the former and the toughness of the latter, with the r^2^ values of 0.85 and 0.82, and the RPDs of 2.9 and 2.4, respectively. According to Williams’s guidelines [31], an r^2^ between 0.83 and 0.90 indicates that the model was usable for most applications, but with caution, including in research. The RPD of 2.4 indicates a poor model which can be used for rough screening; when it is between 2.5 and 2.9, the model is fair and can be used for screening.

The water in whole grain GBR affected its NIR spectrum and the modeling of the textural properties of the cooked rice. As seen in Figure 1, the broad peak of water absorption at 5161 cm^−1^ (1938 nm) due to a combination of vibrations that were due to the O–H stretching + O–H deformation of water [63] was shown, while the peak of amylose was not seen in the spectrum. This was due to the biomaterial contained in the water; the water had a very high NIR radiation absorptivity compared to the other constituents, including amylose in our case. The quantity of amylose in the whole grain GBR analyzed using the colorimetric method was 21.78% [67], and in our experiment, the water content of GBR was only 13–14% wb.

Sampaio et al. [68] showed the pure amylose NIR spectrum, where the major peaks of amylose were at 4633, 4996, 5184, 6834, and 8316 cm^−1^. The model which was best for hardness prediction was developed by using 7506–5446.3 and 4605.4–4242.9 cm^−1^ (1332.3–1836.1 nm and 2171.4–2356.9 nm), which included the amylose vibration band, which was 6834 cm^−1^, while the toughness model was from 9403.8 to 6094.3 cm^−1^ (1063.4–1640.9 nm) and included 6834 and 8316 cm^−1^. Amylose content is correlated with retrogradation behavior, which influences the textural properties of cooked rice [69,70].

Bett-Garber et al. [71] reported that the intensity of the initial starchy coating, slickness, stickiness between grains, cohesiveness, and uniformity of bite of the cooked rice of different long grain rice varieties increased with increasing amounts of water at cooking, whereas hardness, stickiness to lips, springiness, and chewiness decreased.

The amount water used to cook rice was not tested in our experiment. According to Roy et al. [72], the water content of cooked rice ranged from 61% to 69% for Koshihikari (short grain Japonica) and 71% to 72% for IR28 (long grain Indica). Furthermore, Dibba et al. [73] found an average value for the water content of cooked rice of 65.8% and a reasonably broad range of values for individual samples (SD 5.52, *n* = 2666) in prior research in three communities in The Gambia, West Africa.

While amylose as well as water in cooked rice affected its texture, the effect of water in whole grain GBR on the hardness and toughness of cooked rice was similar to the effect of the amylose content. The band of water and starch (6896.6 cm^−1^, which is 1450 nm) was included in the modeling of the optimized model for hardness, and the bands at 8403.4 cm^−1^ (1190 nm) of water and at 6896.6 cm^−1^ (1450 nm) of water and starch were included in the modeling of the optimized model for toughness.

### 3.4. Prediction Performance of PLS Regression Model for Texture of Cooked GBR by Uncooked GBR Grains by MATLAB Using Total Samples

According to the calibration and test set separation using IS, the hardness, toughness, stickiness, and adhesiveness of the cooked GBR were poorly predicted by the PLS regression model using the full wavelength range, and the r^2^ values were 0.26–0.38, 0.38–0.55, 0.07–0.22, and 0.02–0.41, respectively. In the case of the KS separation method, the poor r^2^ values were 0.46–0.60, 0.45–0.56, −0.00–0.27, and 0.05–0.41, respectively. These model predictions were obviously not acceptable.

### 3.5. Prediction Performance of ANN Model for Texture of Cooked GBR by Uncooked GBR Grains by MATLAB Using Total Samples

With the ANN, the model performance was remarkably better than those of PLS regression. Every model using the data from IS and the sorting data separation methods showed an overfitting prediction for every texture parameter, while with KS, the overfitting occurred only with two parameters, except for the hardness and toughness. With CV, the models for toughness, hardness and stickiness were overfitting models (Table 4). The overfitting models provided considerable differences between R^2^ and r^2^, wherever R^2^ was higher and r^2^ was low. In addition, Cawley et al. [74] indicated that the problem of the overfitting model was likely to be the most severe when the sample size was too small and the number of hyperparameters to be tuned was relatively large [75]. Therefore, suitable methods of data separation into a training set and test set and the sample size for modeling were important for obtaining the workable models. In conclusion the stickiness was the most prone to overfitting with the methods used in this study. Therefore, in the case of stickiness, the distribution of the data and the sample size issues must be researched.

The best ANN model for hardness, toughness, and adhesiveness provided R^2^, r^2^, RMSEC, RMSEP, Bias, and RPD values of 1.00, 0.94, 0.10 N, 0.77 N, 0.02 N, and 4.3; 1.00, 0.92, 1.40 Nmm, 9.98 Nmm, 1.6 Nmm, and 3.5; and 0.97, 0.91, 1.35 Nmm, 2.63 Nmm, −0.08 Nmm, and 3.4, respectively (Table 4). Williams et al. [31] indicate that the model can be used for most applications, including quality assurance. When the r^2^ is between 0.92 and 0.96, the model is excellent and can be used for any application; it is very good and can be used for process control and is good and usable for quality control when the RPDs are more than 4.1, 3.5–4.0, and 3.0–3.4 in the case of functionality parameters, which in this case were textural properties.

### 3.6. Comparison of PLS and ANN Model for Texture of Cooked GBR by Uncooked GBR Grains

From the linearized modeling by PLS regression, which dealt with a linear dataset in our case of total samples, the r^2^ for hardness was less than 0.60, but when the ANN was applied, the r^2^ was 0.94, indicating that the relationship between the NIR spectral data and the hardness data of cooked GBR was nonlinear. This was also true for the cases of toughness and adhesiveness (Table 4). ANNs accurately fit nonlinear variables, which is an advantage compared to multivariate linear analysis [76,77]. ANNs have shown their outperformance compared to PLS regression in the estimation of the textural properties of cooked rice by the models developed by using the spectra of whole grain. Aznan et al. [23] used a portable near-infrared spectrometer coupled with an ANN to predict the rice quality traits (color, texture, and pH of cooked rice) of 17 commercial rice types, and a high correlation coefficient (R) of 0.94 was obtained. The prediction of the hardness and toughness of cooked parboiled rice by using the FT-NIR spectra of whole grain parboiled rice combined with PLS regression and principal component regression (PCR) provided the r^2^ values of prediction of 0.70 and 0.66, respectively, which indicated the lower performance of the PLS regression. Sitakalin and Meullenet [78] reported that the sensory texture prediction of cooked rice achieved by an ANN model was superior to that of the PLS regression model [79]. Lu et al. [69] developed the models by using 166 rice flour NIR spectra combined with interval partial least squares (iPLS) and synergy interval PLS (siPLS); they were characterized by the texture-related properties, i.e., the pasting parameters of rice flour, which provided an R between 0.57 and 0.90; however, the ANN provided an R between 0.70 and 0.99 [69].

## 4. Conclusions

With machine learning, the ANN was outperformed in the evaluation of hardness, toughness, and adhesiveness by the back extrusion test of cooked GBR using the SNV, the raw, and the SNV NIR spectra in the range of 4000–12,500 cm^−1^, respectively, of 188 whole grain samples; the calibration sample set was separated from the prediction set by KS; the KS and CV methods, and the developed model developed can be used for any application, process control, and quality assurance, respectively. The best ANN model for hardness, toughness, and adhesiveness provided R^2^, r^2^, RMSEC, RMSEP, Bias, and RPD values of 1.00, 0.94, 0.10 N, 0.77 N, 0.02 N, and 4.3; 1.00, 0.92, 1.40 Nmm, 9.98 Nmm, 1.6 Nmm, and 3.5; and 0.97, 0.91, 1.35 Nmm, 2.63 Nmm, −0.08 Nmm and 3.4, respectively. Though it had the lower performance, the PLS regression of the 64-sample KDML GRB group and the 64-sample GBR group of various varieties provided models for the hardness of the former and the toughness of the latter, which were usable for most applications, but with caution, including in research. The model which was best for hardness prediction was developed by using 7506–5446.3 and 4605.4–4242.9 cm^−1^, which included the amylose vibration band of 6834.0 cm^−1^, while the toughness model was from 9403.8 to 6094.3 cm^−1^, which included the 6834.0 and 8316.0 cm^−1^ vibration bands of amylose, which influenced the texture of the cooked rice. Additionally, the effect of water in whole grain GBR on the hardness and toughness of the cooked rice was similar to the effect of the amylose content. The band of the water and starch (6896.6 cm^−1^, which is 1450 nm) was included in the modeling of the optimized model for hardness, and the bands at 8403.4 cm^−1^ (1190 nm) of the water and at 6896.6 cm^−1^ (1450 nm) of the water and starch were included in the modeling of the optimized model for toughness. The hardness and the toughness of cooked rice can be predicted by NIR spectroscopy using PLS regression; the texture reference test was accurate, as confirmed by the high maximum R^2^ and high R^2^, while the stickiness and adhesiveness could not be predicted due to the low maximum R^2^, indicating the inaccurate measurement of stickiness by the back extrusion test and the uncorrelated linearity of the adhesiveness with the NIR absorption of the samples, even though the maximum R^2^ was the highest.

The hardness of cooked GBR is an important texture parameter and is the first perceived eating quality by cooked rice consumers. Regarding the different consumers in East and Southeast Asia, for example, most consumers like to have soft-textured cooked rice, while in the Middle East, the consumers prefer a harder texture. The toughness and adhesiveness indicated the cohesive and adhesiveness of cooked rice, respectively, and they were the energy needed for the crushed cooked rice grains to be separated from each other and for separating the crushed cooked rice from other material, such as the compression plate, the stirrers in the mixing machine, or the blades of the kneader, respectively. The ANN model combined with the NIR spectra of whole grain GBR for the hardness, toughness, and adhesiveness of cooked GBR should be implemented for practical use in GBR production factories, for the product formulation where different types or varieties of rice were mixed, for the product control, and for the quality assurance with further updating using more samples and several brands to obtain robust models. This study, at the intersection of food science and machine learning, not only enhances our understanding of rice texture but also exemplifies the transformative potential of modern technology in shaping the future of food quality assessment non-destructively, in a way which is rapid, accurate, precise, and environmentally friendly and has a low operating cost. By the conclusion of this exploration, we aspire to unveil a novel approach to the evaluation of the texture of cooked GBR, ushering in a new era of precision and accuracy in the assessment of rice quality.

## Figures and Tables

**Table 1:** The statistics of textural properties of cooked GBR used for modeling by OPUS.

Parameter	Treatment	Total	Cal	Pre	Calibration Set			Prediction Set		
					Range	Mean	SD	Range	Mean	SD
Adhesiveness, Nmm	Condition-adjusted GBR (24 and 48 h soaking)	60	42	18	(−81.15)–(−56.70)	−67.41	6.28	(−77.93)–(−56.99)	−69.09	6.78
Toughness, Nmm		60	42	18	162.32–245.79	201.78	20.50	172.99–245.40	203.87	21.97
Hardness, N		60	42	18	16.55–24.87	20.29	1.85	17.88–23.71	20.86	2.00
Stickiness, N		60	42	18	(−7.67)–(−4.48)	−5.78	0.88	(−7.37)–(−4.65)	−6.15	0.88
Adhesiveness, Nmm	KDML (1–16)	64	46	18	(−78.86)–(−39.13)	−64.22	7.71	(−76.41)–(−40.69)	−62.77	10.58
Toughness, Nmm		64	46	18	109.69–240.80	196.85	25.63	112.22–233.45	193.51	35.98
Hardness, N		64	46	18	11.90–24.52	20.24	2.73	12.11–24.27	19.59	3.62
Stickiness, N		64	46	18	(−7.12)–(−2.84)	−5.15	1.04	(−6.85)–(−3.12)	−4.82	1.13
Adhesiveness, Nmm	Various varieties (17–32)	64	46	18	(−84.93)–(−52.45)	−69.04	8.59	(−83.79)–(−55.22)	−69.44	9.24
Toughness, Nmm		64	46	18	131.94–300.55	197.98	39.34	152.77–276.90	212.36	40.37
Hardness, N		64	46	18	14.23–29.86	20.27	3.33	15.87–27.93	21.93	4.05
Stickiness, N		64	46	18	(−7.20)–(−3.12)	−5.31	0.88	(−6.18)–(−3.18)	−5.08	0.95
Adhesiveness, Nmm	Market (1–32)	128	90	38	(−84.93)–(−39.13))	−66.71	8.54	(−83.79)–(−40.69)	−65.94	10.12
Toughness, Nmm		128	90	38	109.69–300.55	197.80	33.01	112.22–276.90	201.73	38.76
Hardness, N		128	90	38	11.90–29.86	20.27	3.12	12.11–27.93	20.69	3.76
Stickiness, N		128	90	38	(−7.20)–(−2.84)	−5.14	0.95	(−7.12)–(−3.12)	−5.17	1.08
Adhesiveness, Nmm	All samples	188	129	59	(−84.93)–(−39.13)	−67.17	7.80	(−83.79)–(−40.69)	−66.43	9.28
Toughness, Nmm		188	132	56	109.69–300.55	198.20	24.74	112.22–276.90	199.41	36.72
Hardness, N		188	133	55	11.90–29.86	20.16	2.37	12.11–27.93	20.45	3.40
Stickiness, N		188	131	57	(−7.67)–(−2.84)	−5.38	0.93	(−7.67)–(−3.12)	−5.49	1.12

**Table 2:** The statistics of textural properties of cooked GBR samples used for modeling by MATLAB.

		Hardness		Toughness		Stickiness		Adhesiveness	
		Calibration	Prediction	Calibration	Prediction	Calibration	Prediction	Calibration	Prediction
IS	number	150	38	150	38	150	38	150	38
	min	11.90	12.11	109.69	112.22	−7.67	−7.37	−84.93	−83.79
	max	29.86	29.86	300.55	300.55	−2.84	−2.84	−39.13	−39.13
	mean	20.39	20.52	199.75	201.32	−5.39	−5.35	−67.00	−66.69
	SD	3.32	4.51	34.40	46.02	1.10	1.35	9.83	13.68
KS	number	150	38	150	38	150	38	150	38
	min	11.90	15.87	109.69	152.77	−7.67	−7.67	−84.93	−83.79
	max	29.86	29.86	300.55	300.55	−2.84	−3.12	−39.13	−52.45
	mean	20.32	20.79	199.31	203.05	−5.36	−5.48	−66.33	−69.35
	SD	3.27	4.67	34.00	47.23	1.13	1.25	9.97	13.25
sort	number	150	150	150	38	150	38	150	38
	min	11.90	11.90	109.69	112.22	−7.67	−7.37	−84.93	−83.79
	max	29.86	29.86	300.55	275.84	−2.84	−3.12	−39.13	−52.45
	mean	20.46	20.46	200.39	198.77	−5.38	−5.40	−66.83	−67.38
	SD	3.33	3.33	34.39	45.84	1.10	1.34	9.98	13.29
cv	number	151	37	151	37	151	37	151	37
	min	11.90	12.11	109.69	112.22	−7.67	−7.37	−84.93	−83.79
	max	29.86	26.32	300.55	300.55	−2.84	−2.84	−39.13	−39.13
	mean	20.31	20.84	197.92	208.80	−5.41	−5.28	−66.78	−67.59
	SD	3.39	4.33	33.50	48.59	1.09	1.37	9.92	13.57

**Table 3:** The PLS regression result by OPUS software for prediction of textural quality of cooked GBR by using GBR grains spectra.

Treatment	Parameter	Pretreatment	Rank	Wavenumber	Calibration			Prediction			
					R^2^	RMSEC	RPD	r^2^	RMSEP	RPD	Bias
Condition-adjusted GBR (24 and 48 h soaking)	adhesiveness	Constant offset elimination	2	9403.8–6094.3, 5054–4242.9	0.63	3.94	1.64	0.75	3.33	2.13	1.20
	toughness	Constant offset elimination	5	5778–5446.3, 4605.4–4242.9	0.79	10.10	2.17	0.86	8.10	2.73	−2.10
	hardness	no spec	2	6102–5757.3	0.59	1.22	1.55	0.92	0.55	3.95	0.26
	stickiness	no spec	1	8454.9–7498.3, 4605.4–4242.9	0.12	0.84	1.07	0.03	0.84	1.01	−0.19
KDML (1–16)	adhesiveness	first + MSC	9	7506–5446.3	0.84	3.44	2.51	0.74	5.29	2.05	1.64
	toughness	first + MSC	5	9403.8–7498.3, 4605.4–4242.9	0.68	15.50	1.76	0.84	14.00	2.51	1.27
	hardness	SNV	9	7506–5446.3, 4605.4–4242.9	0.87	1.12	2.74	0.85	1.39	2.90	−0.67
	stickiness	first + straight	7	7506–4597.7	0.76	0.55	2.06	0.68	0.63	1.94	0.26
Various varieties (17–32)	adhesiveness	Con off eli	8	7506–6094.3, 5454–4597.7	0.70	5.23	1.81	0.87	3.19	2.91	0.82
	toughness	SNV	9	9403.8–6094.3	0.84	17.40	2.53	0.82	16.90	2.35	2.45
	hardness	MSC	10	9403.8–7498.3, 6102–4597.7	0.97	0.63	5.95	0.32	3.25	1.29	1.13
	stickiness	no spec	7	7506–6094.3, 5029.7–4597.7	0.71	0.52	1.84	0.73	0.48	2.00	−0.14
Market (1–32)	adhesiveness	SNV	5	9403.8–7498.3, 4605.4–4420.3	0.34	7.13	1.23	0.48	7.20	1.49	2.70
	toughness	no spec	10	9403.8–6094.3, 5454–4597.7	0.64	20.90	1.68	0.71	20.50	1.87	1.61
	hardness	MSC	7	9403.8–6094.3, 5454–4597.7	0.50	2.31	1.41	0.61	2.31	1.62	0.33
	stickiness	first + MSC	5	6102–4597.7	0.42	0.77	1.32	0.44	0.80	1.34	−0.07
total sample	adhesive-ness	Min-Max	7	9403.8–7498.3, 4605.4–4242.9	0.52	5.54	1.45	0.21	8.16	1.14	1.33
	toughness	no spec	10	9403.8–7498.3, 6102–5770.3	0.53	20.10	1.46	0.63	22.20	1.69	−5.11
	hardness	SNV	9	9403.8–7498.3, 6102–4597.7	0.55	1.89	1.50	0.56	2.23	1.51	−0.16
	stickiness	SNV	10	9403.8–6094.3, 5454–4597.7	0.53	0.70	1.45	0.21	0.98	1.14	−0.14

**Table 4:** The ANN model for texture of cooked GBR by uncooked GBR grains spectra.

Parameter	Sampling	Pretreatment	Hidden Layer	Calibration		Prediction			
				R^2^	RMSEC	r^2^	RMSEP	RPD	Bias
Adhesiveness (Nmm)	IS	SNV	10	0.92	2.26	0.62	5.62	1.6	0.42
	KS	raw	5	0.75	4.20	0.61	4.49	1.6	−0.51
	sort	raw	5	0.65	4.98	0.39	6.08	1.3	0.27
	CV	SNV	20	0.97	1.35	0.91	2.63	3.4	−0.08
Toughness (Nmm)	IS	raw	10	0.99	2.77	0.52	21.60	1.4	−1.49
	KS	raw	20	1.00	1.40	0.92	9.98	3.5	1.55
	sort	raw	5	0.80	13.46	0.55	22.16	1.5	−4.12
	CV	SNV	25	0.98	3.94	0.87	10.50	2.8	1.09
Hardness (N)	IS	SNV	25	0.73	1.50	0.54	2.01	1.5	−0.28
	KS	SNV	25	1.00	0.10	0.94	0.77	4.3	0.02
	sort	raw	5	0.68	1.63	0.53	2.10	1.5	0.25
	CV	raw	30	1.00	0.16	0.87	1.27	2.8	0.17
Stickiness (N)	IS	SNV	15	1.00	0.05	0.28	0.86	1.2	−0.05
	KS	SNV	5	0.73	0.54	0.61	0.55	1.6	−0.02
	sort	raw	30	0.99	0.12	0.09	0.97	1.1	0.15
	CV	raw	15	0.80	0.44	0.64	0.65	1.7	−0.02

IS, interval sampling; KS, Kennard–Stone; CV, hold-out cross-validation (hold-out CV); sort, sorting.

## Data Availability

Data are contained within the article.

## Supplementary Materials

The following are available online at https://www.mdpi.com/article/10.3390/foods12244516/s1, Supplement Data S1 of separation methods.
